# BayGO: Bayesian analysis of ontology term enrichment in microarray data

**DOI:** 10.1186/1471-2105-7-86

**Published:** 2006-02-23

**Authors:** Ricardo ZN Vêncio, Tie Koide, Suely L Gomes, Carlos A de B Pereira

**Affiliations:** 1 BIOINFO-USP Núcleo de Pesquisas em Bioinformática, Universidade de São Paulo, Rua do Matão 1010, 05508-090 São Paulo, Brazil; 2 Instituto Israelita de Ensino e Pesquisa Albert Einstein, Hospital Israelita Albert Einstein, Av. Albert Einstein 627, 05651-901 São Paulo, Brazil; 3 Departamento de Bioquímica, Instituto de Química, Universidade de São Paulo, Av. Prof. Lineu Prestes 748, 05508-000 São Paulo, Brazil; 4 Departamento de Estatística, Instituto de Matemática e Estatística, Universidade de São Paulo, Rua do Matão 1010, 05508-090 São Paulo, Brazil

## Abstract

**Background:**

The search for enriched (aka over-represented or enhanced) ontology terms in a list of genes obtained from microarray experiments is becoming a standard procedure for a system-level analysis. This procedure tries to summarize the information focussing on classification designs such as Gene Ontology, KEGG pathways, and so on, instead of focussing on individual genes. Although it is well known in statistics that association and significance are distinct concepts, only the former approach has been used to deal with the ontology term enrichment problem.

**Results:**

BayGO implements a Bayesian approach to search for enriched terms from microarray data. The R source-code is freely available at  in three versions: Linux, which can be easily incorporated into pre-existent pipelines; Windows, to be controlled interactively; and as a web-tool. The software was validated using a bacterial heat shock response dataset, since this stress triggers known system-level responses.

**Conclusion:**

The Bayesian model accounts for the fact that, eventually, not all the genes from a given category are observable in microarray data due to low intensity signal, quality filters, genes that were not spotted and so on. Moreover, BayGO allows one to measure the statistical association between generic ontology terms and differential expression, instead of working only with the common significance analysis.

## Background

A systems biology approach which is becoming increasingly used in microarray data analysis is the search for enriched (aka over-represented or enhanced) terms in a list of interesting genes. This kind of approach tries to disclose the biological meaning behind the massive amount of data derived from high-throughput techniques [1,2]. By translating the results into a more human-friendly output, the search for over-represented terms can reveal pathway connections to track biological processes, helping the biologists to build system-level hypotheses.

The problem of ontology term enrichment is generally treated as the test for terms in a gene list that are present in higher numbers than it would be expected only by chance. The terms analyzed are generally derived from an ontology or a classification design. Many genome sequencing projects define their own organism-specific gene classification but recently, we are witnessing an increase in the use of general and standardized vocabularies and classification procedures, such as the one proposed by The Gene Ontology Consortium [3] or by the KEGG database [4].

There are many software packages and web sites addressing the ontology term enrichment problem and it is difficult to acknowledge all of them. The most commonly known are those listed in the Gene Ontology [5] web-site [6], such as: Onto-Express [7], GeneMerge [8], FuncAssociate [9], FatiGO [10], GOstat [11], GOArray [12], GO::TermFinder [13], THEA [14] and OntologyTraverser [15]; but there are other options such as LACK [16], which deals with generic lexical bias. Recently, more than ten different tools were compared and reviewed in [17].

Despite several options, there is no software that searches for enriched ontology terms using a Bayesian statistical framework. Moreover, to our knowledge, all the software packages available are based on the hypothesis test paradigm. We believe that, with the aim of defining enriched terms, the measure of statistical association can be an informative alternative or a useful complement to the usual statistical significance. Thus, our original contributions in this work are: (i) to provide a software that measures the statistical association between differential gene expression and a given ontology term and (ii) to present a statistical model for the enrichment problem that takes into account the realistic fact that, sometimes, not all the genes previously known as related to a given property are in fact observed.

We provide a mathematical source-code that can be easily accommodated into pre-existent software packages or pipelines, in addition to a version for Windows that can be interactively controlled and a web-tool that supports organisms that share special local interest and are neglected by mainstream tools.

## Implementation

### Measure of statistical association

The genes in a microarray dataset can be organized using a classification scheme such as GO [3], KEGG [4] or an organism-specific categorization. Let *i *be the list of genes related to the term(s) in focus. Let *j *be the list of genes related to term(s) different from the term(s) *i*. The dataset is described by a 2 × 3 contingency table:

Differentially expressedNon differentially expressed        i-jijj−iXi−jXijXj−iNi−j−Xi−jNij−XijNj−i−Xj−i
 MathType@MTEF@5@5@+=feaafiart1ev1aaatCvAUfKttLearuWrP9MDH5MBPbIqV92AaeXatLxBI9gBaebbnrfifHhDYfgasaacH8akY=wiFfYdH8Gipec8Eeeu0xXdbba9frFj0=OqFfea0dXdd9vqai=hGuQ8kuc9pgc9s8qqaq=dirpe0xb9q8qiLsFr0=vr0=vr0dc8meaabaqaciaacaGaaeqabaqabeGadaaakeaafaqaaeWabaaabaaabaGaeeiraqKaeeyAaKMaeeOzayMaeeOzayMaeeyzauMaeeOCaiNaeeyzauMaeeOBa4MaeeiDaqNaeeyAaKMaeeyyaeMaeeiBaWMaeeiBaWMaeeyEaKNaeeiiaaIaeeyzauMaeeiEaGNaeeiCaaNaeeOCaiNaeeyzauMaee4CamNaee4CamNaeeyzauMaeeizaqgabaGaeeOta4Kaee4Ba8MaeeOBa4MaeeiiaaIaeeizaqMaeeyAaKMaeeOzayMaeeOzayMaeeyzauMaeeOCaiNaeeyzauMaeeOBa4MaeeiDaqNaeeyAaKMaeeyyaeMaeeiBaWMaeeiBaWMaeeyEaKNaeeiiaaIaeeyzauMaeeiEaGNaeeiCaaNaeeOCaiNaeeyzauMaee4CamNaee4CamNaeeyzauMaeeizaqMaeeiiaaIaeeiiaaIaeeiiaaIaeeiiaaIaeeiiaaIaeeiiaaIaeeiiaaIaeeiiaacaauaabeqadmaaaeaacqWGPbqAcqGGTaqlcqWGQbGAaeaacqWGPbqAcqWGQbGAaeaacqWGQbGAcqGHsislcqWGPbqAaeaacqWGybawdaWgaaWcbaGaemyAaKMaeyOeI0IaemOAaOgabeaaaOqaaiabdIfaynaaBaaaleaacqWGPbqAcqWGQbGAaeqaaaGcbaGaemiwaG1aaSbaaSqaaiabdQgaQjabgkHiTiabdMgaPbqabaaakeaacqWGobGtdaWgaaWcbaGaemyAaKMaeyOeI0IaemOAaOgabeaakiabgkHiTiabdIfaynaaBaaaleaacqWGPbqAcqGHsislcqWGQbGAaeqaaaGcbaGaemOta40aaSbaaSqaaiabdMgaPjabdQgaQbqabaGccqGHsislcqWGybawdaWgaaWcbaGaemyAaKMaemOAaOgabeaaaOqaaiabd6eaonaaBaaaleaacqWGQbGAcqGHsislcqWGPbqAaeqaaOGaeyOeI0IaemiwaG1aaSbaaSqaaiabdQgaQjabgkHiTiabdMgaPbqabaaaaaaa@AF44@

**Table 1:** Gene Ontology terms considered significantly enriched (*P *< 0.05) by the Bayesian approach. The GO terms marked with an asterisk are all those considered significant (*p*-value < 0.05) by the Frequentist approach. G is the gamma measure of statistical association and G90% is its 90% credibility interval ("error-bar")

ID	Description	*P*	*G*	*G*_90%_
GO:0006986	response to unfolded protein *	0.000	1.00	[0.95; 1.00]
GO:0006457	protein folding *	0.000	0.86	[0.76; 0.91]
GO:0051082	unfolded protein binding *	0.000	0.83	[0.74; 0.88]
GO:0004252	serine-type endopeptidase activity	0.005	0.85	[0.69; 0.94]
GO:0004222	metalloendopeptidase activity	0.005	0.72	[0.56; 0.84]
GO:0005515	protein binding *	0.010	0.80	[0.65; 0.89]
GO:0031072	heat shock protein binding	0.015	0.81	[0.78; 0.84]
GO:0008233	peptidase activity	0.015	0.63	[0.50; 0.81]
GO:0006508	proteolysis and peptidolysis	0.020	0.59	[0.41; 0.73]
GO:0016702	oxidoreductase activity, acting on single donors	0.020	0.81	[0.78; 0.83]
GO:0004176	ATP-dependent peptidase activity	0.025	1.00	[0.80; 1.00]
GO:0009377	HslUV protease activity	0.025	1.00	[1.00; 1.00]
GO:0030163	protein catabolism	0.025	0.81	[0.79; 0.84]
GO:0004295	trypsin activity	0.030	1.00	[1.00; 1.00]
GO:0015969	guanosine tetraphosphate metabolism	0.030	1.00	[1.00; 1.00]
GO:0019836	hemolysis	0.030	1.00	[1.00; 1.00]
GO:0006886	intracellular protein transport	0.045	0.66	[0.62; 0.70]

where: *X*_*i*-*j *_is the number of differentially expressed genes that are *i*-exclusive, *N*_*i*-*j *_is the total number of genes that are *i*-exclusive, *X*_*ij *_is the number of differentially expressed genes belonging to the *i *and *j *intersection, *N*_*ij *_is the total number of genes belonging to the *i *and *j *intersection, *X*_*j*-*i *_is the number of differentially expressed genes that are not related to the term *i *(thus *j*-exclusive) and *N*_*j*-*i *_is the total number of genes that are not related to the term *i *(thus *j*-exclusive). Note that a given gene must be counted in one and only one of these cases and that summing over all *N*. yields the total number of considered genes.

In 1954, Goodman and Kruskal wrote a classical work discussing several issues on measures of association for contingency tables [18]. We choose to report the Goodman and Kruskal's gamma [19] since it is symmetric and normalized between -1 and 1. The gamma measure (*G*) is a 2 × 3 extension of the known 2 × 2 Yule's *Q*:

*G *= (p - q)/(p + q)     (1)

where p = *X*_*i*-*j*_(*N*_*ij *_- *X*_*ij *_+ *N*_*j*-*i *_- *X*_*j*-*i*_) + *X*_*ij*_(*N*_*j*-*i *_- *X*_*j*-*i*_) and q = *X*_*j*-*i*_(*N*_*ij *_- *X*_*ij *_+ *N*_*i*-*j *_- *X*_*i*-*j*_) + *X*_*ij*_(*N*_*i*-*j *_- *X*_*i*-*j*_). *G *values near 1 indicate that the property described by the term(s) in focus have an important role in the biological phenomena studied. *G *values below or near zero have no relevant biological interpretation since they mean the absence of association with differential gene expression.

### Probabilistic model and bayesian inference

Focussing on a given term(s) and using the same notation from the previous sub-section, let *x*_*i*-*j *_be the number of differentially expressed genes observed that are *i*-exclusive, *n*_*i*-*j *_be the total number of observed genes that are *i*-exclusive, *x*_*ij *_is the number of observed differentially expressed genes belonging to the *i *and *j *intersection, *n*_*ij *_is the total number of observed genes belonging to the *i *and *j *intersection, *x*_*j*-*i *_is the number of differentially expressed genes observed that are not related to the term *i *and *n*_*j*-*i *_is the total number of genes that are not related to the term *i*. Note that *N*. ≥ *n*. ≥ *x*. for any sub-index, represented by the "dot".

It is possible to realize that *x*.|*X*.,*N*.,*n*. follows a hypergeometric distribution [20]:

Pr(x.=k|X.,N.,n.)=(X.k)(N.−X.n.−k)(N.n.)−1
 MathType@MTEF@5@5@+=feaafiart1ev1aaatCvAUfKttLearuWrP9MDH5MBPbIqV92AaeXatLxBI9gBaebbnrfifHhDYfgasaacH8akY=wiFfYdH8Gipec8Eeeu0xXdbba9frFj0=OqFfea0dXdd9vqai=hGuQ8kuc9pgc9s8qqaq=dirpe0xb9q8qiLsFr0=vr0=vr0dc8meaabaqaciaacaGaaeqabaqabeGadaaakeaaieqacqWFqbaucqWFYbGCcqGGOaakcqWG4baEcqGGUaGlcqGH9aqpcqWGRbWAcqGG8baFcqWGybawcqGGUaGlcqGGSaalcqWGobGtcqGGUaGlcqGGSaalcqWGUbGBcqGGUaGlcqGGPaqkcqGH9aqpdaqadaqaauaabeqaceaaaeaacqWGybawcqGGUaGlaeaacqWGRbWAaaaacaGLOaGaayzkaaWaaeWaaeaafaqabeGabaaabaGaemOta4KaeiOla4IaeyOeI0IaemiwaGLaeiOla4cabaGaemOBa4MaeiOla4IaeyOeI0Iaem4AaSgaaaGaayjkaiaawMcaamaabmaabaqbaeqabiqaaaqaaiabd6eaojabc6caUaqaaiabd6gaUjabc6caUaaaaiaawIcacaGLPaaadaahaaWcbeqaaiabgkHiTiabigdaXaaaaaa@58AF@

We will omit *N*. and *n*. from the notation since they are always known numbers. Using a non-informative uniform as the *a priori *distribution for *X*. and the hypergeometric likelihood above, it is possible to show that the *a posteriori *distribution of (*X*. - *x*.)|*x*. is a BetaBinomial distribution [20]:

Pr(X.−x.=k|x.)=(N.−n.k)B(k+1;N.−n.−k+1)
 MathType@MTEF@5@5@+=feaafiart1ev1aaatCvAUfKttLearuWrP9MDH5MBPbIqV92AaeXatLxBI9gBaebbnrfifHhDYfgasaacH8akY=wiFfYdH8Gipec8Eeeu0xXdbba9frFj0=OqFfea0dXdd9vqai=hGuQ8kuc9pgc9s8qqaq=dirpe0xb9q8qiLsFr0=vr0=vr0dc8meaabaqaciaacaGaaeqabaqabeGadaaakeaaieqacqWFqbaucqWFYbGCcqGGOaakcqWGybawcqGGUaGlcqGHsislcqWG4baEcqGGUaGlcqGH9aqpcqWGRbWAcqGG8baFcqWG4baEcqGGUaGlcqGGPaqkcqGH9aqpdaqadaqaauaabeqaceaaaeaacqWGobGtcqGGUaGlcqGHsislcqWGUbGBcqGGUaGlaeaacqWGRbWAaaaacaGLOaGaayzkaaGaemOqaiKaeiikaGIaem4AaSMaey4kaSIaeGymaeJaei4oaSJaemOta4KaeiOla4IaeyOeI0IaemOBa4MaeiOla4IaeyOeI0Iaem4AaSMaey4kaSIaeGymaeJaeiykaKcaaa@5624@

where *B *is the beta special function.

Therefore, the 2 × 3 contingency table can be rewritten as:

Differentially expressedNon differentially expressed        i-jijj−ixi−j+(Xi−j−xi−j)|xi−jxij+(Xij−xij)|xijxj−i+(Xj−i−xj−i)|xj−iNi−j−xi−j−(Xi−j−xi−j)|xi−jNij−xij−(Xij−xij)|xijNj−i−xj−i−(Xj−i−xj−i)|xj−i
 MathType@MTEF@5@5@+=feaafiart1ev1aaatCvAUfKttLearuWrP9MDH5MBPbIqV92AaeXatLxBI9gBaebbnrfifHhDYfgasaacH8akY=wiFfYdH8Gipec8Eeeu0xXdbba9frFj0=OqFfea0dXdd9vqai=hGuQ8kuc9pgc9s8qqaq=dirpe0xb9q8qiLsFr0=vr0=vr0dc8meaabaqaciaacaGaaeqabaqabeGadaaakeaafaqaaeWabaaabaaabaGaeeiraqKaeeyAaKMaeeOzayMaeeOzayMaeeyzauMaeeOCaiNaeeyzauMaeeOBa4MaeeiDaqNaeeyAaKMaeeyyaeMaeeiBaWMaeeiBaWMaeeyEaKNaeeiiaaIaeeyzauMaeeiEaGNaeeiCaaNaeeOCaiNaeeyzauMaee4CamNaee4CamNaeeyzauMaeeizaqgabaGaeeOta4Kaee4Ba8MaeeOBa4MaeeiiaaIaeeizaqMaeeyAaKMaeeOzayMaeeOzayMaeeyzauMaeeOCaiNaeeyzauMaeeOBa4MaeeiDaqNaeeyAaKMaeeyyaeMaeeiBaWMaeeiBaWMaeeyEaKNaeeiiaaIaeeyzauMaeeiEaGNaeeiCaaNaeeOCaiNaeeyzauMaee4CamNaee4CamNaeeyzauMaeeizaqMaeeiiaaIaeeiiaaIaeeiiaaIaeeiiaaIaeeiiaaIaeeiiaaIaeeiiaaIaeeiiaacaauaabeqadmaaaeaacqWGPbqAcqGGTaqlcqWGQbGAaeaacqWGPbqAcqWGQbGAaeaacqWGQbGAcqGHsislcqWGPbqAaeaacqWG4baEdaWgaaWcbaGaemyAaKMaeyOeI0IaemOAaOgabeaakiabgUcaRmaabmaabaGaemiwaG1aaSbaaSqaaiabdMgaPjabgkHiTiabdQgaQbqabaGccqGHsislcqWG4baEdaWgaaWcbaGaemyAaKMaeyOeI0IaemOAaOgabeaaaOGaayjkaiaawMcaamaaeeaabaGaemiEaG3aaSbaaSqaaiabdMgaPjabgkHiTiabdQgaQbqabaaakiaawEa7aaqaaiabdIha4naaBaaaleaacqWGPbqAcqWGQbGAaeqaaOGaey4kaSYaaeWaaeaacqWGybawdaWgaaWcbaGaemyAaKMaemOAaOgabeaakiabgkHiTiabdIha4naaBaaaleaacqWGPbqAcqWGQbGAaeqaaaGccaGLOaGaayzkaaWaaqqaaeaacqWG4baEdaWgaaWcbaGaemyAaKMaemOAaOgabeaaaOGaay5bSdaabaGaemiEaG3aaSbaaSqaaiabdQgaQjabgkHiTiabdMgaPbqabaGccqGHRaWkdaqadaqaaiabdIfaynaaBaaaleaacqWGQbGAcqGHsislcqWGPbqAaeqaaOGaeyOeI0IaemiEaG3aaSbaaSqaaiabdQgaQjabgkHiTiabdMgaPbqabaaakiaawIcacaGLPaaadaabbaqaaiabdIha4naaBaaaleaacqWGQbGAcqGHsislcqWGPbqAaeqaaaGccaGLhWoaaeaacqWGobGtdaWgaaWcbaGaemyAaKMaeyOeI0IaemOAaOgabeaakiabgkHiTiabdIha4naaBaaaleaacqWGPbqAcqGHsislcqWGQbGAaeqaaOGaeyOeI0YaaeWaaeaacqWGybawdaWgaaWcbaGaemyAaKMaeyOeI0IaemOAaOgabeaakiabgkHiTiabdIha4naaBaaaleaacqWGPbqAcqGHsislcqWGQbGAaeqaaaGccaGLOaGaayzkaaWaaqqaaeaacqWG4baEdaWgaaWcbaGaemyAaKMaeyOeI0IaemOAaOgabeaaaOGaay5bSdaabaGaemOta40aaSbaaSqaaiabdMgaPjabdQgaQbqabaGccqGHsislcqWG4baEdaWgaaWcbaGaemyAaKMaemOAaOgabeaakiabgkHiTmaabmaabaGaemiwaG1aaSbaaSqaaiabdMgaPjabdQgaQbqabaGccqGHsislcqWG4baEdaWgaaWcbaGaemyAaKMaemOAaOgabeaaaOGaayjkaiaawMcaamaaeeaabaGaemiEaG3aaSbaaSqaaiabdMgaPjabdQgaQbqabaaakiaawEa7aaqaaiabd6eaonaaBaaaleaacqWGQbGAcqGHsislcqWGPbqAaeqaaOGaeyOeI0IaemiEaG3aaSbaaSqaaiabdQgaQjabgkHiTiabdMgaPbqabaGccqGHsisldaqadaqaaiabdIfaynaaBaaaleaacqWGQbGAcqGHsislcqWGPbqAaeqaaOGaeyOeI0IaemiEaG3aaSbaaSqaaiabdQgaQjabgkHiTiabdMgaPbqabaaakiaawIcacaGLPaaadaabbaqaaiabdIha4naaBaaaleaacqWGQbGAcqGHsislcqWGPbqAaeqaaaGccaGLhWoaaaaaaa@2751@

The degree of association determined in Eq.1 is now a function of the random variables *X*_*i*-*j*_, *X*_*ij *_and *X*_*j*-*i*_, described by the BetaBinomial *a posteriori *probability functions.

In computational terms, it means that several independent 2 × 3 contingency tables are built using random variates drawn according to their respective BetaBinomial probability distributions. The empirical probability distribution of the association level is obtained by applying Eq.1 to each of these random contingency tables. The significance analysis and the construction of credibility intervals ("errorbars") are based on this empirical distribution of *G*.

We have used 90% credibility intervals as default, but this parameter can be easily adjusted in the source-code. The credibility intervals are defined as previously described [21].

### Assessing the significance of association measurements

To evaluate the significance of a measured statistical association, we have determined the probability *P *= **Pr**(*G*_*M *_≥ *G*_*obs*_), where *G*_*obs *_is the observed association and *G*_*M *_is the association obtained from MonteCarlo simulations. This simulation process accounts for the ontology's structure since it is influenced by the connectivity of the ontology graph.

The simulation consists in uniformly sampling lists from the observed genes. In each simulation round, this random list of length *M *is considered as the list of differentially expressed genes. Ideally, the number of genes used in each round considers that there is an intrinsic error when one defines a gene as differentially expressed. Keeping the length constant *M *= *n*_*i*-*j *_+ *n*_*ij *_+ *n*_*j*-*i *_could be an unrealistic approximation, due to the possibility of a global false-calling rate. We used a generic and heuristic sampling rule considering this length as *M *~ Poisson(*n*_*i*-*j *_+ *n*_*ij *_+ *n*_*j*-*i*_). It is possible to incorporate other rules to define the length *M *in BayGO source-code. The rule can be derived from the particular method used for the identification of differentially expressed genes.

The definition of *P *is similar to the Frequentist *p*-value if *G*_*obs *_is a real number and *M *is kept constant. It is known that, in this configuration, simulations tend to agree with the theoretical results from Fisher-like methods. However, if we consider that not all the genes were observed and thus, the association is also a random variable, then the Frequentist *p*-value is not defined. On the other hand, the Bayesian analog *P *is still valid since the event {*G*_*M *_≥ *G*} is well defined. This probability is calculated in the BayGO software by counting the number of times that this event occurs during the MonteCarlo rounds. Note that, in each round, a new *G *value is calculated based on random variates drawn according to the BetaBinomial model described in the previous sub-section.

### BayGO source-code

BayGO source-code is provided in three versions: Linux, Windows^® ^and as a package for building a custom web-based interface. The core of BayGO is written as R language scripts [22]. The package for web-based interfaces is a set of Perl, HTML and R source-codes.

The main source-code of BayGO receives as inputs: an R binary "database" file, a list of differentially expressed genes in a plain-text file, a list of genes that were not differentially expressed and the number of simulation rounds desired for the significance and error-bar determination. The R binary "database" file keeps the organism information: an ontology table, containing all available ontology terms and descriptions, and an ontology-to-gene table, listing all the genes classified under each term. This file is built only once by an auxiliary script and it is used in several analysis. To build this "database" it is necessary to provide as inputs: a gene-to-ontology plain-text file (similar to the GO .goa files, for example) and an ontology description plain-text file. Detailed examples are provided in the BayGO user-manual available at BayGO Home-page [23].

BayGO assumes that the ontology-to-gene or gene-to-ontology tables are adequately built by the user, considering the different levels of abstraction of the GO graph desired. These issues include taking into account the hierarchical structure when considering father/sons ontology terms and defining an arbitrary truncation level of the graph [17].

The Linux version was designed to be easily incorporated into pre-existing tools or pipelines. There are several software packages that deal with the ontology term enrichment problem [17] and many of them are integrated with relevant databases, creating a knowledge discovery environment for the biologist user. We did not try to surpass these efforts and therefore, we provide a software that receives and exports simple plain-text files and is operated in a non-interactive form. These features allow BayGO to be incorporated as a mathematical module in complex and complete pipelines. An example of an OpenSource software which has a powerful GUI is Clutree [24].

The version for Windows^® ^was designed to be extremely user-friendly. It does not require any programming skills and it is controlled interactively by menus. The functions to analyze the data and build R binary "database" files are integrated in the windows-style menus.

Finally, the web-based version was designed to allow the researchers to establish their own local web-tool for the ontology term enrichment analysis. It is easy to realize that the organisms supported by the web-based tools are restricted to those with a vast research community such as human, mouse, yeast and so on. This version will enable research communities focussed on other organisms to settle a useful resource, assuming a simple infrastructure such as a web-server with PERL/CGI allowed.

### Microarray experiments

To test the BayGO software, we used microarray data from the heat shock response in bacteria. Since this stress triggers a conserved biological response from a system-level viewpoint, we believe that meaningful results could show BayGO usefulness. Therefore, we analyzed the transcriptional response of the phytopathogenic bacterium *Xylella fastidiosa *exposed to a temperature shift-up from 29°C to 40°C for 25 minutes.

The complete dataset and all the MIAME-required details regarding the construction, hybridization, image acquisition and expression ratio normalization are publicly available at GEO database [25,26] under the accession number GSE3044. The microarray slides and data were obtained according to [27]. The expression ratio between the 40°C and 29°C (control) conditions was normalized using the LOWESS fitting exactly as in [27].

Unreliable spots, presenting intensities too similar to the local background or saturated were filtered out. Spots presenting mean signal intensity below the mean background plus 2 times its standard deviation in Cy3 and Cy5 simultaneously were eliminated from subsequent analysis. Saturated spots were also discarded. The HTself method [28] was used to define the differentially expressed and non differentially expressed genes. Briefly, the HTself method uses self-self experiments (cDNA from the 29°C control condition labeled independently with both Cy3 and Cy5 fluorescent dye and hybridized simultaneously in the same microarray slide) to derive an intensity-dependent cutoff curve. We used credibility intervals of 0.99, window size of 1.0 and window step of 0.2. It is assumed that genes found consistently outside this cutoff curve are differentially expressed. A gene was classified as differentially expressed or not if it has at least 5 measured replicates and 80% of the replicates are outside or inside the intensity-dependent cutoff curves, respectively.

The pathway information about the bacterium *Xylella fastidiosa *was obtained directly from the KEGG web-site [29,4] and the Gene Ontology information from the GOA@EBI web-site [5,30].

We considered an ontology term as significantly enriched if it presents a *p*-value smaller than 0.05. To reach the conclusions using the Frequentist method, we adopted the default parameters in GeneMerge [8] software, including Bonferroni correction and using the arrayed genes as the reference set. To reach the conclusions using our Bayesian method, we used the whole-genome as the reference set, as required by our proposed model.

## Results and discussion

### Measurement of statistical association in addition to statistical significance

The measurement of association is an extensively studied issue in statistics [18,19,31]. The usual meaning of "association" refers to coefficients that measure the strength of relationships; they are used to measure the relationship when there is a dichotomy. Two other kinds of association measurements are the well-known "correlation", which is used when both variables are intervals, and the "reliability", which is used to analyze a variable with itself. The concept of statistical association is different from statistical significance. Measures of significance compare the strength of an observed relationship with one that would be expected by chance, if one performs random sampling. The significance analysis is influenced not only by the strength of a relationship but also by other parameters such as the sample size or the sampling procedure. It is possible to have a relationship that presents a non-significant strong association or a significant weak relationship. Significance is relevant only when one has a random sample whereas association is always relevant to draw conclusions. Since significance and association are not equivalent, researchers may report both measures when discussing their findings. The use of association measurements for the problem of ontology term enrichment in bioinformatics has not been explored yet; the predominance of significance approaches is evident.

We propose to measure the statistical association in the context of the ontology term enrichment problem using the Goodman and Kruskal's gamma factor *G *[19] (Eq.1). *G *is a symmetric index, normalized between -1 and 1. The interpretation of this measure is that *G *values near 1 indicate strong positive association. The positive association of a given term with differential gene expression means that the property described by the ontology term on focus can be involved in the biological phenomenon studied. Negative and zero association are biologically meaningless in this context. The zero value means that there is no statistical association and the negative values indicate association with non-differential gene expression.

It is not possible to define in advance a cutoff value above which one can consider an ontology term as presenting a relevant association. On the other hand, there are some traditionally used cutoffs for significance such as 0.01 or 0.05. It is intuitive to rely on a conclusion showing significance of 0.05, but it is difficult to foresee the value of association that represents a strong association in a general context (the same argument holds for correlation measurements). Depending on the application, the value of 0.95 can represent a strong association (or correlation); in other context, the same value can be an insufficient evidence of association between two facts. Ideally, sorting ontology terms by their *G *values and performing external independent biological assays could provide the association cutoffs. In order to evaluate the significance of the obtained associations, we calculated the probability *P *= **Pr**(*G*_*M *_≥ *G*), where *G*_*obs *_is the observed association and *G*_*M *_is a random variable obtained by MonteCarlo simulation (see *Implementation *section for details).

### Bayesian model accounting for non-observed genes

In a typical microarray dataset, a gene could not be detected for several reasons: it was not spotted on the array due to a selection procedure; it did not meet the quality standards on intensity, integrity, technical reproducibility, etc. If we cannot observe all the genes, the number of genes differentially expressed observed is an estimate and, therefore, we need an inferential framework.

The advantages and disadvantages of the Bayesian statistics over the Frequentist approach belong to an old and endless debate in statistics. Some advantages of the Bayesian approach for microarray analysis are well discussed elsewhere [32]. In the problem of ontology term enrichment, the Bayesian framework allows the incorporation of prior information regarding the known size of each set of genes associated to an ontology term.

If we consider that all genes associated to a certain term were observed, the association measure *G *is a real number. In addition, if the significance analysis is made upon random lists of differentially expressed genes with the same length of the observed differentially expressed gene list, the probability *P *= **Pr**(*G*_*M *_≥ *G*) is equivalent to the usual Frequentist *p*-value. These assumptions are tacitly used when one performs all the Fisher-like tests [17].

However, if one cannot observe all the genes associated to a term, then the association should be treated as a random variable whose probability distribution reflects our ignorance about the true value. Our ignorance becomes smaller as we observe more genes. Moreover, we can establish credibility intervals ("error-bars") for the degree of association, similarly as discussed previously in a SAGE analysis context [21]. The Bayesian analogue for the *p*-value, *P *= **Pr**(*G*_*M *_≥ *G*_*obs*_), is still valid even if both *G*_*M *_and *G*_*obs *_are random variables, whereas the Frequentist *p*-value is not defined in this case.

It is possible to show that, under certain conditions, once one observes a portion of the differentially expressed genes related to a given ontology term, the unknown remainder portion is described by a BetaBinomial probability distribution [20]. This result holds under a non-informative uniform *a priori *(see *Implementation *section for mathematical details) given the number of differentially and non differentially expressed genes that were observed and the number of genes known to be related to the ontology term. In other words, as it is performed in an electoral process, we estimate the behavior of the finite population of genes related to an ontology term using a sample of it.

### Examples of the BayGO usefulness

To show the usefulness of the BayGO software, we devise some illustrative examples of situations that one can face when analyzing microarray data. One of the examples shows the usefulness of the statistical association and the other examples show the Bayesian feature of considering the sampling effect.

To highlight the difference between our Bayesian method and one that does not account for the sampling effect, we have arbitrarily chosen the "hypergeometric" test to represent the Frequentist Fisher-like approaches [8,17].

We will consider a fictitious test-organism with characteristics compatible with several real organisms. Suppose that this test-organism has 4000 genes, classified into several categories that are described by ontology terms. Let us focus on an arbitrary class and list all the genes related to a term *i *that describes this class. The size of the set *i *is 30, and only 5 genes are exclusively related to the property described by the term *i*. Let *j *be the list of genes that have other terms different from *i *related to them. The size of this set *j *is 3975. The set *i-j *contains the genes that are exclusively related to the ontology term *i *while the set *j-i *contains the genes that are not related to the ontology term *i*. The set *ij *contains the genes that are not *i *exclusive. Finally, suppose that we carried out microarray experiments comprising all the known genes.

The gene-to-ontology table for this test-organism, the complete Bayesian and Frequentist results and the corresponding gene lists used in the following illustrative examples are available as Additional File 1.

In the first example, suppose that all the 4000 genes are observable and the microarray experiment yielded 400 differentially expressed genes, distributed as follows:

Differentially expressedNon differentially expressed        i-jijj−i317380283590
 MathType@MTEF@5@5@+=feaafiart1ev1aaatCvAUfKttLearuWrP9MDH5MBPbIqV92AaeXatLxBI9gBaebbnrfifHhDYfgasaacH8akY=wiFfYdH8Gipec8Eeeu0xXdbba9frFj0=OqFfea0dXdd9vqai=hGuQ8kuc9pgc9s8qqaq=dirpe0xb9q8qiLsFr0=vr0=vr0dc8meaabaqaciaacaGaaeqabaqabeGadaaakeaafaqaaeWabaaabaaabaGaeeiraqKaeeyAaKMaeeOzayMaeeOzayMaeeyzauMaeeOCaiNaeeyzauMaeeOBa4MaeeiDaqNaeeyAaKMaeeyyaeMaeeiBaWMaeeiBaWMaeeyEaKNaeeiiaaIaeeyzauMaeeiEaGNaeeiCaaNaeeOCaiNaeeyzauMaee4CamNaee4CamNaeeyzauMaeeizaqgabaGaeeOta4Kaee4Ba8MaeeOBa4MaeeiiaaIaeeizaqMaeeyAaKMaeeOzayMaeeOzayMaeeyzauMaeeOCaiNaeeyzauMaeeOBa4MaeeiDaqNaeeyAaKMaeeyyaeMaeeiBaWMaeeiBaWMaeeyEaKNaeeiiaaIaeeyzauMaeeiEaGNaeeiCaaNaeeOCaiNaeeyzauMaee4CamNaee4CamNaeeyzauMaeeizaqMaeeiiaaIaeeiiaaIaeeiiaaIaeeiiaaIaeeiiaaIaeeiiaaIaeeiiaaIaeeiiaacaauaabeqadmaaaeaacqWGPbqAcqGGTaqlcqWGQbGAaeaacqWGPbqAcqWGQbGAaeaacqWGQbGAcqGHsislcqWGPbqAaeaacqaIZaWmaeaacqaIXaqmcqaI3aWnaeaacqaIZaWmcqaI4aaocqaIWaamaeaacqaIYaGmaeaacqaI4aaoaeaacqaIZaWmcqaI1aqncqaI5aqocqaIWaamaaaaaa@8D60@

In this 1^st ^scenario, there is no qualitative difference between the two approaches. The *p*-value obtained by the Frequentist test is <10^-13^, indicating a significant enrichment of the term *i*. Accordingly, using the Goodman and Kruskal's gamma (Eq.1) to measure the statistical association between the term *i *and differential expression, one obtains *G*_*obs *_= 0.899, a value close to 1 which means strong association. Moreover, since all available genes were observed, *G *is known without uncertainty and its 90% credibility interval [0.899; 0.899] has zero length. The MonteCarlo simulations with 1000 rounds indicate significant association since *P *= 0.

Suppose that, for the same dataset, the result from half of the genes related to term *i *was not measured by typical reasons such as: it did not meet quality standards on intensity, integrity, technical reproducibility, and so on. Now the contingency table is:

Differentially expressedNon differentially expressed        i-jijj−i19380143590
 MathType@MTEF@5@5@+=feaafiart1ev1aaatCvAUfKttLearuWrP9MDH5MBPbIqV92AaeXatLxBI9gBaebbnrfifHhDYfgasaacH8akY=wiFfYdH8Gipec8Eeeu0xXdbba9frFj0=OqFfea0dXdd9vqai=hGuQ8kuc9pgc9s8qqaq=dirpe0xb9q8qiLsFr0=vr0=vr0dc8meaabaqaciaacaGaaeqabaqabeGadaaakeaafaqaaeWabaaabaaabaGaeeiraqKaeeyAaKMaeeOzayMaeeOzayMaeeyzauMaeeOCaiNaeeyzauMaeeOBa4MaeeiDaqNaeeyAaKMaeeyyaeMaeeiBaWMaeeiBaWMaeeyEaKNaeeiiaaIaeeyzauMaeeiEaGNaeeiCaaNaeeOCaiNaeeyzauMaee4CamNaee4CamNaeeyzauMaeeizaqgabaGaeeOta4Kaee4Ba8MaeeOBa4MaeeiiaaIaeeizaqMaeeyAaKMaeeOzayMaeeOzayMaeeyzauMaeeOCaiNaeeyzauMaeeOBa4MaeeiDaqNaeeyAaKMaeeyyaeMaeeiBaWMaeeiBaWMaeeyEaKNaeeiiaaIaeeyzauMaeeiEaGNaeeiCaaNaeeOCaiNaeeyzauMaee4CamNaee4CamNaeeyzauMaeeizaqMaeeiiaaIaeeiiaaIaeeiiaaIaeeiiaaIaeeiiaaIaeeiiaaIaeeiiaaIaeeiiaacaauaabeqadmaaaeaacqWGPbqAcqGGTaqlcqWGQbGAaeaacqWGPbqAcqWGQbGAaeaacqWGQbGAcqGHsislcqWGPbqAaeaacqaIXaqmaeaacqaI5aqoaeaacqaIZaWmcqaI4aaocqaIWaamaeaacqaIXaqmaeaacqaI0aanaeaacqaIZaWmcqaI1aqncqaI5aqocqaIWaamaaaaaa@8C66@

In this 2^nd ^scenario, both approaches are still indicating a significant enrichment of the term *i*. However, the statistical association measured is now a random variable, since the data is a sample from the complete scenario. The Frequentist test's *p*-value is 3.5·10^-4^. The counts for all *i*-related genes were diminished proportionally (e.g. approximately half), thus, the measured association remains the same *G*_*obs *_= 0.899. Nevertheless, there is now an uncertainty related to the measured statistical association and its 90% credibility interval is [0.829;0.937]. Despite the uncertainty, the association measured is much more probable than the association obtained from 1000 uniformly generated random lists of differentially expressed genes, since *P *= 0.

Now, suppose that only one fifth of the *i *related genes had their expression status defined. The contingency table is:

Differentially expressedNon differentially expressed        i-jijj−i04380023590
 MathType@MTEF@5@5@+=feaafiart1ev1aaatCvAUfKttLearuWrP9MDH5MBPbIqV92AaeXatLxBI9gBaebbnrfifHhDYfgasaacH8akY=wiFfYdH8Gipec8Eeeu0xXdbba9frFj0=OqFfea0dXdd9vqai=hGuQ8kuc9pgc9s8qqaq=dirpe0xb9q8qiLsFr0=vr0=vr0dc8meaabaqaciaacaGaaeqabaqabeGadaaakeaafaqaaeWabaaabaaabaGaeeiraqKaeeyAaKMaeeOzayMaeeOzayMaeeyzauMaeeOCaiNaeeyzauMaeeOBa4MaeeiDaqNaeeyAaKMaeeyyaeMaeeiBaWMaeeiBaWMaeeyEaKNaeeiiaaIaeeyzauMaeeiEaGNaeeiCaaNaeeOCaiNaeeyzauMaee4CamNaee4CamNaeeyzauMaeeizaqgabaGaeeOta4Kaee4Ba8MaeeOBa4MaeeiiaaIaeeizaqMaeeyAaKMaeeOzayMaeeOzayMaeeyzauMaeeOCaiNaeeyzauMaeeOBa4MaeeiDaqNaeeyAaKMaeeyyaeMaeeiBaWMaeeiBaWMaeeyEaKNaeeiiaaIaeeyzauMaeeiEaGNaeeiCaaNaeeOCaiNaeeyzauMaee4CamNaee4CamNaeeyzauMaeeizaqMaeeiiaaIaeeiiaaIaeeiiaaIaeeiiaaIaeeiiaaIaeeiiaaIaeeiiaaIaeeiiaacaauaabeqadmaaaeaacqWGPbqAcqGGTaqlcqWGQbGAaeaacqWGPbqAcqWGQbGAaeaacqWGQbGAcqGHsislcqWGPbqAaeaacqaIWaamaeaacqaI0aanaeaacqaIZaWmcqaI4aaocqaIWaamaeaacqaIWaamaeaacqaIYaGmaeaacqaIZaWmcqaI1aqncqaI5aqocqaIWaamaaaaaa@8C54@

In this 3^rd ^scenario, the two approaches clearly disagree. The Frequentist test's *p*-value is 0.32 indicating no significant enrichment. The association measure gives the same result *G*_*obs *_= 0.899 with the 90% credibility interval [0.724;0.948]. The significance of the obtained association is *P *= 0.04, indicating that the enrichment could be accepted in face of the usual 0.05 cutoff.

Intuitively, we believe that finding an abundance of 4/400 for the differentially expressed genes is relevant when comparing with the total abundance of 6/3976 for all genes. Therefore, we believe that the association measurement is capturing the essential feature of the data, even if subjected to uncertainty.

In the following, we will consider another extreme example: when the observed genes are highly biased. Toward this aim, suppose that the test-organism has a gene annotation slightly different from the one used above, allocating the same 30 genes in a different way among the ontology terms. In the previous example, we emphasized the consistent pattern of the association measurement if the observed sample is "equilibrated" over the possible results. Now, using the same test-organism and the same microarray setup, suppose that we could observe all available genes, yielding the following contingency table:

Differentially expressedNon differentially expressed        i-jijj−i053952503575
 MathType@MTEF@5@5@+=feaafiart1ev1aaatCvAUfKttLearuWrP9MDH5MBPbIqV92AaeXatLxBI9gBaebbnrfifHhDYfgasaacH8akY=wiFfYdH8Gipec8Eeeu0xXdbba9frFj0=OqFfea0dXdd9vqai=hGuQ8kuc9pgc9s8qqaq=dirpe0xb9q8qiLsFr0=vr0=vr0dc8meaabaqaciaacaGaaeqabaqabeGadaaakeaafaqaaeWabaaabaaabaGaeeiraqKaeeyAaKMaeeOzayMaeeOzayMaeeyzauMaeeOCaiNaeeyzauMaeeOBa4MaeeiDaqNaeeyAaKMaeeyyaeMaeeiBaWMaeeiBaWMaeeyEaKNaeeiiaaIaeeyzauMaeeiEaGNaeeiCaaNaeeOCaiNaeeyzauMaee4CamNaee4CamNaeeyzauMaeeizaqgabaGaeeOta4Kaee4Ba8MaeeOBa4MaeeiiaaIaeeizaqMaeeyAaKMaeeOzayMaeeOzayMaeeyzauMaeeOCaiNaeeyzauMaeeOBa4MaeeiDaqNaeeyAaKMaeeyyaeMaeeiBaWMaeeiBaWMaeeyEaKNaeeiiaaIaeeyzauMaeeiEaGNaeeiCaaNaeeOCaiNaeeyzauMaee4CamNaee4CamNaeeyzauMaeeizaqMaeeiiaaIaeeiiaaIaeeiiaaIaeeiiaaIaeeiiaaIaeeiiaaIaeeiiaaIaeeiiaacaauaabeqadmaaaeaacqWGPbqAcqGGTaqlcqWGQbGAaeaacqWGPbqAcqWGQbGAaeaacqWGQbGAcqGHsislcqWGPbqAaeaacqaIWaamaeaacqaI1aqnaeaacqaIZaWmcqaI5aqocqaI1aqnaeaacqaIYaGmcqaI1aqnaeaacqaIWaamaeaacqaIZaWmcqaI1aqncqaI3aWncqaI1aqnaaaaaa@8D60@

It is clear that, from both Frequentist test and Bayesian model, there is no significant enrichment of the term *i*, since the *p*-value is 0.17, *G*_*obs *_= 0.282 without uncertainty and *P *= 0.11.

Suppose that one cannot observe the result from the genes related exclusively to the term *i*. Let us consider, for instance, that the 25 *i*-exclusive genes were not spotted on the microarray slide due to gene selection. Therefore, the contingency table is:

Differentially expressedNon differentially expressed        i-jijj−i05395003575
 MathType@MTEF@5@5@+=feaafiart1ev1aaatCvAUfKttLearuWrP9MDH5MBPbIqV92AaeXatLxBI9gBaebbnrfifHhDYfgasaacH8akY=wiFfYdH8Gipec8Eeeu0xXdbba9frFj0=OqFfea0dXdd9vqai=hGuQ8kuc9pgc9s8qqaq=dirpe0xb9q8qiLsFr0=vr0=vr0dc8meaabaqaciaacaGaaeqabaqabeGadaaakeaafaqaaeWabaaabaaabaGaeeiraqKaeeyAaKMaeeOzayMaeeOzayMaeeyzauMaeeOCaiNaeeyzauMaeeOBa4MaeeiDaqNaeeyAaKMaeeyyaeMaeeiBaWMaeeiBaWMaeeyEaKNaeeiiaaIaeeyzauMaeeiEaGNaeeiCaaNaeeOCaiNaeeyzauMaee4CamNaee4CamNaeeyzauMaeeizaqgabaGaeeOta4Kaee4Ba8MaeeOBa4MaeeiiaaIaeeizaqMaeeyAaKMaeeOzayMaeeOzayMaeeyzauMaeeOCaiNaeeyzauMaeeOBa4MaeeiDaqNaeeyAaKMaeeyyaeMaeeiBaWMaeeiBaWMaeeyEaKNaeeiiaaIaeeyzauMaeeiEaGNaeeiCaaNaeeOCaiNaeeyzauMaee4CamNaee4CamNaeeyzauMaeeizaqMaeeiiaaIaeeiiaaIaeeiiaaIaeeiiaaIaeeiiaaIaeeiiaaIaeeiiaaIaeeiiaacaauaabeqadmaaaeaacqWGPbqAcqGGTaqlcqWGQbGAaeaacqWGPbqAcqWGQbGAaeaacqWGQbGAcqGHsislcqWGPbqAaeaacqaIWaamaeaacqaI1aqnaeaacqaIZaWmcqaI5aqocqaI1aqnaeaacqaIWaamaeaacqaIWaamaeaacqaIZaWmcqaI1aqncqaI3aWncqaI1aqnaaaaaa@8C64@

In this scenario, one is led to believe that there is a significant enrichment, since all spotted genes related to the term *i *were found to be differentially expressed. In fact, the *p*-value is <10^-5 ^and the association is very strong with *G*_*obs *_= 1. However, the 90% credibility interval of the association measurement is large [0.382;1.00] and the significance is low with *P *= 0.34.

We believe that this example shows the importance of considering the sampling effect in the significance or in the association result, manifested by the large "error-bar".

### Application to real microarray data

To validate the BayGO approach, we used the microarray experiment data corresponding to a relatively well-characterized biological phenomenon: the heat shock response in bacteria. The high temperature stress is an aggressive environmental perturbation for bacteria, triggering system level changes in their transcriptional program. Although this stress response has many peculiarities depending on the particular bacteria studied, there are known conserved responses that can guide the validation of our model.

In this sense, we analyzed the response of the phytopathogenic bacterium *Xylella fastidiosa *to a temperature shift-up from 29°C to 40°C for 25 minutes, using DNA microarrays fully described in the GEO database under the accession number GSE3044. To define the up-regulated and down-regulated genes, as well the genes whose expression was unchanged, we used the HTself method [28] (see more details in the *Implementation *section).

In this example, we will focus on the up-regulated genes induced by the temperature stress; therefore, the non-induced genes are the down-regulated plus the non-perturbed genes. It is not our aim in this work to explore the particular biological implications of this dataset. The complete study of heat shock response comprising several time-points, along with the biological rationale and conclusions will appear elsewhere (T. Koide, R.Z.N. Vêncio and S.L.Gomes, submitted).

We avoid making extensive comparisons with the methods available since there are several combinations of ways in which a term enrichment analysis can be performed [17]. A given method can yield a different qualitative result depending on the multiple-test correction used, depending on several free parameters or depending on the input ontology (for example, GO analysis can be performed considering or not certain connections of the graph). Furthermore, we believe that presently, there is no reasonable independent methodology of biological validation to confront the system-level biological conclusions obtained with different softwares.

We chose to present the BayGO results along with the results obtained by GeneMerge [8]. GeneMerge is one of the few methods available that accepts arbitrary ontologies and supports arbitrary organisms, not being limited to the most studied ones [17]. Note, however, that this is an illustrative comparison.

The first analysis is relative to KEGG pathways. Additional File 2 contains the complete output from the Bayesian and Frequentist softwares for KEGG terms analysis. The pathway terms significantly enriched (*p*-value < 0.05) according to the Frequentist methods are: *Protein folding and associated processing *and *Folding, Sorting and Degradation*. The terms enriched (*P *< 0.05) according to our Bayesian methods are: *Protein folding and associated processing*, *Folding, Sorting and Degradation *and *Genetic Information Processing*. These results are completely compatible with what is known about the bacterial heat shock response [33]. It seems that BayGO correctly captured the KEGG term *Genetic Information Processing *since it is known that bacteria facing such stress change their gene expression program in a broad system-level sense.

The second analysis is relative to Gene Ontology. Additional File 2 contains the outputs from the BayGO and GeneMerge softwares for GO terms analysis. The ontology terms significantly enriched (*p*-value < 0.05) according to the Frequentist method are: *Protein folding *(*GO:0006457*), *Unfolded protein binding *(*GO:0051082*), *Response to unfolded protein *(*GO:0006986*) and *Protein binding *(*GO:0005515*). The terms enriched according to our Bayesian method (*P *< 0.05) compose a more numerous set, including the 4 terms found by the Frequentist method. Table 1 shows these significant terms.

The biological insight provided by the Bayesian method seems to be richer when one is trying to elaborate a system-level picture of the transcriptional disturbance caused by heat shock. For instance, BayGO was able to highlight the term *HslUV protease activity *(*GO:0009377*) and *Heat shock protein binding *(*GO:0031072*) that were not highlighted by the Frequentist analysis. Both activities are classically known to be involved in the heat shock response [33].

We restricted our illustrative comparisons to the significance analysis since there is no similar software that approaches the statistical association issue presented in this work. We believe that the use of association measurements can help the biologists in their microarray analysis. For instance, the term *Chaperone activator activity *(*GO:0030189*) presents a strong association level (*G*_*obs *_= 1) but not a high significance (*P *< 0.17) and the chaperone molecules are known to be related to the heat shock response. Results for all terms are available in Additional File 2.

It is important to note that the comparison performed in this section should be carefully considered since there are many possible variations for the Frequentist analysis [17]. However, we believe that these experimental results show the usefulness of BayGO software.

### BayGO source-code

Our source-code is provided as R language [22] scripts in three versions: Linux, Windows. and as a package for building custom web-based interface locally. The Linux version receives its parameters non-interactively by command-line arguments and thus, it can be easily incorporated into pre-existing pipelines or web-based tools. The version for Windows is a user-friendly program that is controlled interactively by menus, without the necessity of programming, being more appropriate for end-users. Finally, the web-based version is a set of R, Perl and HTML source-codes that allows one to build his own web-based tool locally, focussing on the organisms of interest.

Using the web-based version, we have created a useful web-tool that supports organisms of interest to our local research community [23]. The ever-growing list of supported organisms includes some of those that are neglected by the most used similar tools, such as *Xylella fastidiosa*, *Xanthomonas citri*, *Blastocladiella emersonii*, etc. Although BayGO was designed based on Gene Ontology terms, the software can also be used with other classification designs. For this, the user has to provide a gene-to-ontology hash table for all known genes and not only for those spotted in the microarray slide.

### Caveats, limitations and recommendations

The approach of finding enriched ontology terms in microarray data has several known intrinsic limitations. In our opinion, the most severe limitations are: (i) the practical difficulty in the experimental validation of the conclusions derived from such methods, since it is difficult to manipulate a large set of genes to observe which system-level deductions are real or methods' artifacts; (ii) the large quantity of "free parameters" that can be changed in routinely use of these methods, from the hierarchical structure of ontologies limited by the user to the choice of cutoff *p*-values; (iii) the tacit assumption that a given function, pathway, etc., is "important" or not solely based on numerical aspects (number of genes, statistical association, etc) ignoring the qualitative aspects involved (few differentially expressed genes in a pathway might be sufficient to trigger system-level responses); and (iv) the results rely heavily on gene annotation provided for the studied organism.

It is likely that, even for well studied prokaryotes, many genes have no known function and others are likely to be involved in different processes and therefore, they are associated to multiple presently unassigned ontology terms. A potential implication is that the contingency tables probably hold more uncertainty than it is taken into account by the present models. There are some efforts in trying to quantify the effects of mis-annotation or to establish some form of probabilistic gene annotation, including Bayesian models [34,35]. Future directions of works in the ontology term enrichment problem should include such gene annotation uncertainties.

The ontology term enrichment problem is not restricted to differential gene expression analysis or to microarray derived data. The current models, including the one implemented in BayGO, are capable of analysing the enrichment relative to other dichotomous properties such as "belonging to a particular gene cluster" vs. "not belonging", in clustering analysis [24]; "it is expressed (ON)" vs. "it is not expressed (OFF)", in gene transcription analysis; "narrowly expressed transcripts" vs. "prevalently expressed transcripts" [36]; and so on.

Particularly for BayGO, the dichotomous properties are defined in the two rows of the contingency table. The significance of the statistical association is measured relative to the first row, however, since gamma (Eq.1) is symmetric around zero, flipping rows order will yield the "opposite" analysis. In the illustration presented in the section *Application to real microarray data*, we chose to analyze the induced genes after temperature stress, defining the dichotomous property as "up-regulated" vs. "not up-regulated" (this class includes not differentially expressed and down-regulated genes). Conversely, one may want to focus on the repressed genes defining the contigency table's rows as "down-regulated" vs. "not down-regulated". Another possibility is to analyze "perturbed" vs "no change", independent of the direction of the change.

Generally, the methods to find enriched terms work using nominal lists of genes without knowledge of how the list was obtained. The means by which these gene lists were obtained define the interpretation and meaning of the result. It is important to note that the method used to obtain the differentially expressed genes (or any other property being studied) has an important impact on the enrichment analysis since they determine the gene list that is inputed. In this work, we analysed our microarray data using our recently published HTself method [28] but we provide the same analysis using widely used approaches as well at the Supplemental material [23].

BayGO was designed to be a mathematical module and therefore, it has a limited interactive potential. For instance, it does not provide a graphical user interface (GUI) that allows the user to browse his/her results. These features are complex and are beyond BayGO's scope. It was designed to be incorporated into pre-existing software with elaborated GUI front-end. One good option of such front-end is the OpenSource software Clutree [24]. One of the future directions for BayGO evolution could be its incorporation on available GUIs.

## Conclusion

When dealing with the system-level problem of finding enriched terms from microarray data, most of the softwares available use only the significance analysis. In this work, we have introduced the use of a measure of statistical association between ontology terms and differentially expressed genes, in addition to the common significance analysis. We elaborated a Bayesian statistical model for the ontology term enrichment problem that incorporates information about the composition of the ontology by taking into account genes that were not observed in the microarray data. The examples given in this work aim at making the investigators aware of the necessity of considering the sampling problem when drawing system-level conclusions.

The BayGO software can be used with generic gene-to-ontology tables and not only with GO classification. The web-tool is implemented for a set of organisms with particular interest to our local research community, but we made available the source-code that allows one to build custom web-tools for other organisms. We also made available an R source-code and a web-based tool that calculates these association measurements and their significances.

Finally, we would like to highlight that the estimation of statistical association and statistical significance are not equivalent procedures. We believe that the use of statistical association should be more explored in bioinformatics. Statistical association is an established tool in statistics, widely used in other research fields since it allows the visualization of relationships inside the data that are not considered when using only significance analysis

## Availability and requirements

• Project name: BayGO

• Project home page: 

• Operating system(s): Platform independent (Linux, Windows, Mac OS X and web-service for supported organisms)

• Programming language: R.

• Other requirements: to build a local version of the web-service it is necessary to have Apache with CGI allowed and Perl.

• License: under the GNU General Public License

## List of abbreviations

GO: Gene Ontology

KEGG: Kyoto Encyclopedia of Genes and Genomes

SAGE: Serial Analysis of Gene Expression

GUI: graphical user interface

## Authors' contributions

RZNV and TK conducted the work and drafted the manuscript. RZNV and CABP are responsible for the statistical model and software. TK and SLG are responsible for all the experimental work and biological interpretation. All the authors wrote and approved the manuscript.

## Supplementary Material

Additional File 1 Zipped file containing all the data relative to the artificial test-organism. It contains: the gene-to-GO table, the complete genome, the input data for creating the examples of "Examples of the BayGO usefulness" section and all the outputs/results obtained for those examples. Click here for file

Additional File 2 Excel or Gnumeric worksheet containing all the results from term enrichment analysis of Xylella fastidiosa heat shock experiment, described in the "Application to real microarray data" section. Click here for file
